# Interpretable deep learning-based hierarchical multi-modal fusion model for predicting HER2 expression in gastric cancer

**DOI:** 10.3389/fonc.2026.1745228

**Published:** 2026-05-18

**Authors:** Chenxi Hu, Changfeng Feng, Ziyi Ye, Shuaihang Kong, Jing Han, Tingting Yang, Kai Zhong, Xingguang Zhong, Qijun Shen

**Affiliations:** 1 The Fourth School of Clinical Medicine, Zhejiang Chinese Medical University, Hangzhou First People's Hospital, Hangzhou, Zhejiang, China; 2 Department of Radiology, Affiliated Hangzhou First People's Hospital, School of Medicine, Westlake University, Hangzhou, Zhejiang, China; 3 Department of Radiology, Hangzhou Children’s Hospital, Hangzhou, Zhejiang, China; 4 Department of Radiology, Zhejiang Kangjing Hospital, Hangzhou, Zhejiang, China; 5 Department of Radiology, Zhejiang Cancer Hospital, Hangzhou, Zhejiang, China

**Keywords:** algorithm, endoscopy, ErbB receptors, gastric cancer, tomography X-ray computed

## Abstract

**Objectives:**

Aimed to develop and evaluate a hierarchical multimodal framework to improve HER2 status prediction in Gastric Cancer (GC) patients.

**Methods:**

This retrospective study included 402 endoscopically identified GC patients (2014–2023), among whom 92 had pathologically confirmed HER2 status. Endoscopic images were analyzed using ResNet-50 to extract deep learning (DL) features and to develop a separate model for predicting invasion depth to aid in HER2 prediction. Radiomic features were extracted from contrast-enhanced CT images using Pyradiomics, with optimal features selected based on reproducibility (ICC > 0.75) and feature importance. Six machine learning algorithms were used to build multimodal models, and performance metrics such as area under the curve (AUC) and sensitivity were evaluated. Additionally, SHapley Additive exPlanations (SHAP) values were used to assess the contributions of individual features within the best-performing model.

**Results:**

Using ResNet-50 analysis of endoscopic images, an invasion depth prediction model was developed (validation AUC: 0.79; 95% CI: 0.77-0.81). The probability output from this model was integrated as a variable and combined with three optimal DL endoscopic features (1, 2, 8) for HER2 prediction. Additionally, seven discriminative features were extracted via radiomic analysis. When integrated with endoscopic, radiomics and clinical data, the multimodal model consistently outperformed unimodal approaches, with Logistic Regression yielding the highest validation AUC (0.83, 95% CI: 0.74-0.91). SHAP analysis highlighted Wavelength-HHL_GLSZM_SAHGE and DLfeature_8 as key HER2 predictors.

**Conclusion:**

By integrating clinical, endoscopic, and radiomic data, the hierarchical multimodal approach enhances HER2 expression prediction in GC, thereby optimizing clinical decision-making, and enabling tailored HER2-targeted therapies.

## 1 Introduction

Gastric cancer (GC) is one of the most prevalent and lethal malignancies worldwide, particularly in East Asia (1, 2). HER2 has emerged as a crucial biomarker for GC, as its overexpression is associated with tumor aggressiveness (3) and responsiveness to targeted therapies such as trastuzumab (4, 5). Accurate evaluation of HER2 status is critical for selecting appropriate treatment strategies and improving patient outcomes. Currently, HER2 assessment primarily relies on invasive methods, including immunohistochemistry (IHC) and fluorescence *in situ* hybridization (FISH) (6). However, these methods have notable limitations, such as dependence on tissue samples obtained through biopsy or surgery, susceptibility to sampling errors, high costs, and time-consuming processes. These drawbacks highlight the urgent need for alternative approaches to facilitate early and reliable HER2 evaluation in clinical practice.

Gastroscopy remains the gold standard for GC diagnosis by enabling real-time mucosal visualization. However, its diagnostic accuracy is constrained by operator expertise and subjectivity. With the advancement of artificial intelligence (AI) technologies, particularly convolutional neural networks (CNNs), deep learning models have demonstrated strong performance in predicting tumor invasion depth from endoscopic images (7–9). This is clinically significant because invasion depth is a key determinant of clinical staging and treatment decision-making in gastric cancer, deeper tumor invasion is generally linked to more aggressive biological behavior (10), which previous studies suggest may correlate with HER2 expression patterns (11, 12). Beyond depth prediction, CNNs have also been applied to extract high-level visual features from endoscopic images that encode tumor morphology and tissue characteristics, serving as objective, data-driven representations of tumor biology for downstream classification tasks (13).

While endoscopy captures high-resolution surface details, it cannot assess the deeper tumor microenvironment. Contrast-enhanced CT offers a non-invasive, comprehensive view of tumor morphology, vascularity and staging (14–16). However, traditional CT assessment relies on subjective qualitative evaluation. To improve objectivity, radiomics—an emerging quantitative imaging analysis technique—can be applied. By extracting high-dimensional quantitative features from standard imaging modalities, radiomics provides comprehensive information about tumor microenvironments and molecular characteristics (17–19).

Integrating multimodal data can reveal critical diagnostic features often obscured in single-modality analyses, providing a more holistic understanding of disease complexity (20–22). In the context of GC, different diagnostic tools capture distinct yet complementary biological dimensions. Specifically, while endoscopic AI offers crucial superficial mucosal details and invasion depth estimates, CT radiomics reveals the deeper macroscopic microenvironment, and clinical data provide essential patient baselines. Recognizing this synergy, our study aims to develop a hierarchical multimodal fusion model to improve HER2 expression prediction. By systematically integrating these features, this approach replaces subjective, experience-based methods with a comprehensive quantitative analysis, ultimately aiming to optimize clinical workflows and enable personalized treatment strategies.

## 2 Materials and methods

### Ethics statement

This retrospective study was approved by the Medical Ethics Committee of our institution (IRB approval number: 2025ZN053-1; Date: 6 March 2025) and was conducted in accordance with relevant guidelines. Informed consent was waived.

### Patient cohort

**Figure 1:**
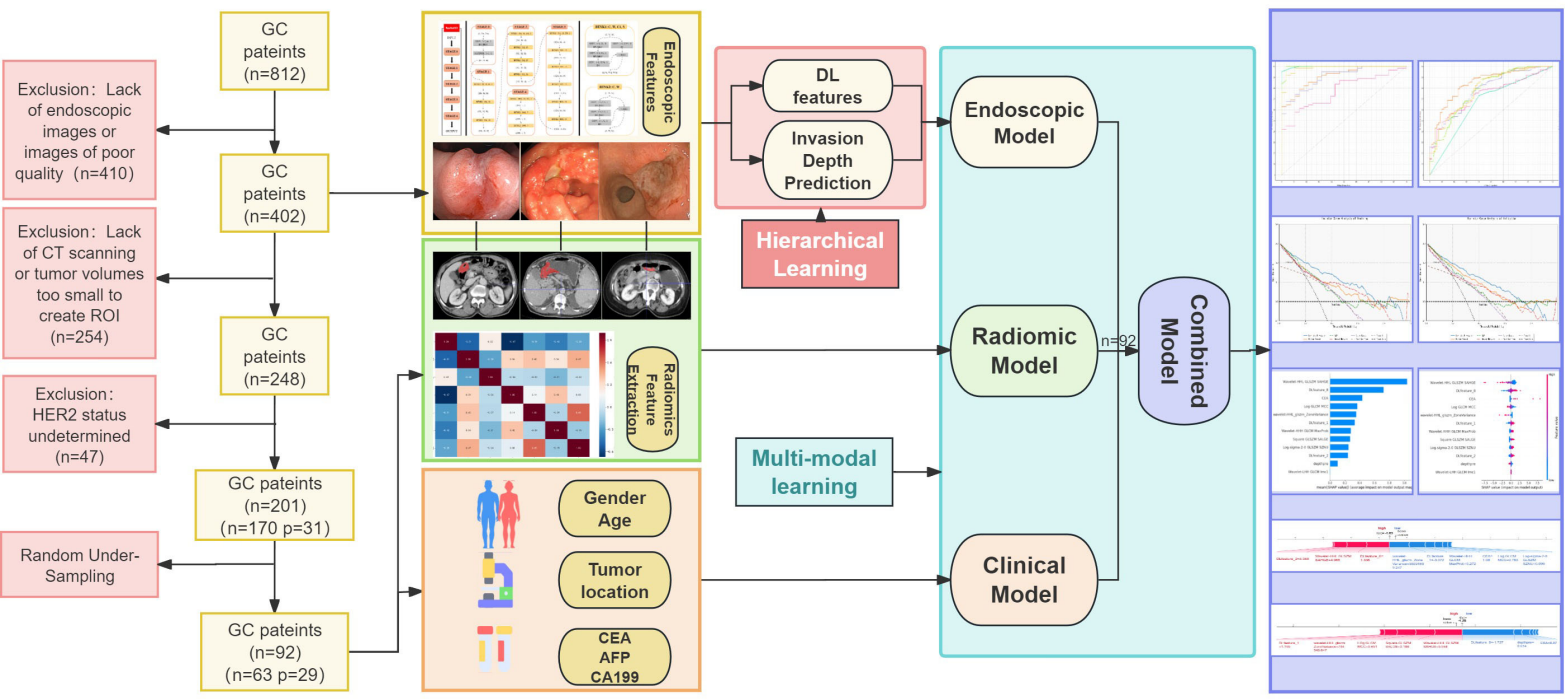
Workflow.

Retrospective data from 812 pathologically confirmed GC patients at a medical center between January 2014 and August 2023 were analyzed. Through strict exclusion criteria, 402 patients were first used to establish an endoscopic prediction model, and ultimately 201 patients were eligible for combined modeling. Due to inherent class imbalance in real-world clinical data, Random Under-Sampling was applied, yielding a final balanced cohort of 92 patients. The inclusion criteria were as follows: (1) histopathologically confirmed GC following surgery; (2) HER2 status determined via IHC or IHC and FISH; (3) availability of high-quality CT scans and endoscopic images. The exclusion criteria were as follows: (1) patients with incomplete clinical or imaging data; (2) poor gastric distension or inadequate image quality for diagnostic evaluation; (3) tumor volumes too small to locate the primary lesion on CT images; (4) patients with a history of preoperative chemotherapy, radiotherapy, neoadjuvant therapy, or remnant GC. The workflow of the analysis is summarized in **Figure 1**.

### HER2 status ascertainment

HER2 status was determined using IHC staining of tumor tissue samples. HER2 positivity was defined as an IHC score of 3+ (uniform intense membranous staining in >10% of tumor cells) or an IHC score of 2+ with confirmation of HER2 gene amplification by FISH, in accordance with established guidelines (23). Tumors with IHC scores of 0 or 1+ were classified as HER2-negative. HER2 testing was performed by a pathologist with 15 years of experience, blinded to clinical and imaging data to ensure unbiased assessment.

### Data collection

For each patient, endoscopic images and CT scans were collected. Endoscopic images were captured using standard upper gastrointestinal endoscopes (GIF-H290Z, GIF-Q260J, GIF-HQ290, EG-601WR, EG-760R and EG-760Z; Olympus Medical Systems, Tokyo, Japan). The GC images were captured with standard white light imaging (WLI). Images demonstrating inadequate luminal distension, post-biopsy hemorrhage, highlight saturation, motion blur, defocus artifacts, or excessive mucus retention were systematically excluded.

The CT scans were carried out on a uCT710 60-slice scanner. Scanning parameters included a tube voltage of 120 kV, a tube current of automatic milliampere-second modulation (Auto mAs), a field of view of 40 cm, and a matrix size of 512 × 512. The scan range extended from the dome of the diaphragm to the inferior border of the pubic symphysis, with a thickness of 5 mm per layer. After injection of 1.5 mL/kg of iodinated contrast material with an automatic power pump injector at a rate of 3.0 mL/s into the antecubital vein, CT images in the arterial and portal venous phase were respectively obtained at a 30 s and 70 s delay after infusion of contrast material.

Demographic data and clinical characteristics, including age, gender, tumor location, tumor size, CA199, CEA and AFP, were retrospectively collected from electronic medical records by a 5-year-experienced gastroenterologist.

### Endoscopic feature extraction and DL model development

To ensure data consistency across the cohort, all endoscopic images underwent manual quality control during data curation. Poor-quality or excessively noisy images were excluded, and only lesion-containing images with adequate visualization were retained for downstream analysis. All included images then underwent standardized preprocessing. Specifically, each image was resized to 224 × 224 pixels and normalized using the ImageNet mean ([0.485, 0.456, 0.406]) and standard deviation ([0.229, 0.224, 0.225]) to standardize the input distribution for the pre-trained network.

During the training phase, this was followed by data augmentation methods including random horizontal flipping, rotation, and color jittering (brightness, contrast, saturation, hue) with 0.5 probability. To address class imbalance, class weights were calculated and incorporated into the loss function, and the Synthetic Minority Over-sampling Technique (SMOTE) was applied exclusively to the training set after data splitting to generate additional synthetic samples for the minority class, while the validation set was kept unchanged to avoid data leakage. The model was trained using a stochastic gradient descent (SGD) optimizer with momentum = 0.9, L2 regularization = 1e-4, and a learning rate of 0.0002 over 70 epochs.

In the invasion depth prediction task, a total of 2, 631 endoscopic images from 402 gastric cancer (GC) patients were included. To prevent data leakage arising from multiple images per patient, the dataset was split at the patient level into an 8:2 ratio for training and validation. Specifically, all images from the same patient were assigned exclusively to either the training or the validation cohort. These were composed of 1, 869 and 762 images of early and advanced GC, respectively. The reference standard for invasion depth labeling was based on postoperative histopathological assessment. Based on postoperative histopathological confirmation, lesions confined to the mucosa or submucosa were classified as superficial (early) invasion, whereas those extending into the muscularis propria, subserosa, or beyond were categorized as deep invasion. These pathological classifications followed the criteria of the Japanese Classification of Gastric Carcinoma (3rd English Edition) (24) and the 8th edition of the AJCC TNM staging system (10). The final fully connected layer of ResNet50 was replaced with a binary classifier (mucosal/submucosal vs. deeper invasion). The model was fine-tuned end-to-end using class-weighted cross-entropy loss, with validation performance monitored through the receiver operating characteristic curve (ROC) area under the curve (AUC). Early stopping was applied if validation loss plateaued for 10 consecutive epochs, and the best model was selected based on the highest validation AUC.

For deep learning (DL) feature extraction, the pre-trained ResNet50 backbone (convolutional layers) was frozen, and region of interest (ROI) images were fed into the network to extract 2048-dimensional features from the global average pooling layer (avgpool). These DL features were aggregated by averaging probabilities across all endoscopic images per patient, followed by principal component analysis (PCA) to reduce dimensionality from 2048 to 32. The reduced features were subsequently used for HER2 status analysis. The complete code for model training and deep learning feature extraction is publicly available at https://github.com/1059685121-png/Endoscopic-Feature-Extraction-.

### Radiomic feature extraction and selection

Enhanced CT scans were retrieved from the picture archiving and communication system (PACS) and transferred to ITK-SNAP software (version 3.6.0, University of Pennsylvania). Initial manual segmentation of gastric tumor regions was performed by a board-certified radiologist (reader 1, 5-year experience), creating three-dimensional volumes of interest (VOIs) through volumetric reconstruction. To evaluate interobserver agreement of radiomic features, a random subset of 30 cases underwent repeat segmentation three months later by a senior abdominal radiologist (reader 2, 10-year expertise), forming reproducibility assessment datasets. Prior to radiomic feature extraction, all images underwent standardized preprocessing to ensure feature reproducibility across patients and scanners. Specifically, CT volumes were resampled to isotropic voxel spacing of 1.0 × 1.0 × 1.5 mm³, and voxel intensities were normalized using z-score normalization within a fixed Hounsfield unit range of −200 to 300 HU. Gray-level discretization was performed with a bin width of 25 HU. Radiomic features were extracted from contrast-enhanced CT scans using Pyradiomics, a standardized platform. Features were derived from regions of interest (ROIs) segmented manually to encompass the entire tumor volume. Feature selection was performed in three steps: (1) filtering features with low variance; (2) eliminating features with high collinearity (correlation coefficient > 0.9); and (3) ranking features based on importance using gradient-boosting decision trees, where feature importance was quantified by the mean decrease in impurity (MDI) criterion via the feature_importances_ attribute in scikit-learn.

### Model development and validation

To integrate data across different modalities, we employed an early feature-level fusion strategy. Specifically, the selected features from distinct modalities—DL features refined via LASSO regression, radiomic features retained after variance thresholding (intraclass correlation coefficient > 0.75) and importance ranking, invasion depth probability derived from the ResNet50 classifier, and statistically significant clinical indicators—were directly concatenated into a unified feature vector for each patient prior to model training. Before entering the validation loop, all features were standardized to have zero mean and unit variance using global StandardScaler, thereby eliminating dimensional discrepancies.

To maximize data utilization and ensure a robust evaluation, model performance was assessed using leave-one-out cross-validation. In each iteration, one patient was held out as the test sample and the remaining patients were used for model training. Six machine learning algorithms (Decision Tree, Logistic Regression (LR), Naive Bayes, Support Vector Machine, Random Forest, and Neural Network) were implemented in Orange (v3.39.0; scikit-learn backend) to construct the multimodal classification models. Default hyperparameters were used for all algorithms, and the key hyperparameters are summarized in **Supplementary Table 1**.

Performance metrics, including AUC, accuracy, sensitivity, specificity, precision, and F1 score, were calculated and visualized using radar plots. Decision curve analysis (DCA) was performed to evaluate clinical utility across probability thresholds. Using the optimal algorithm identified from initial comparisons, we constructed distinct models for individual modalities (Endoscopic, Clinical, Radiomic) and the combined features. Finally, SHapley Additive exPlanations (SHAP) were applied to quantify the feature contributions and interpret the decision-making process of the optimal model trained on the entire dataset.

### Statistical analysis

All statistical analyses were performed using SPSS software (Version 25.0; IBM Corp., Armonk, NY, USA). Continuous variables were tested for normality using the Kolmogorov-Smirnov test and compared using independent sample t-tests or Mann-Whitney U tests as appropriate. Categorical variables were analyzed using chi-square or Fisher’s exact tests, with P-values < 0.05 considered statistically significant. Multivariate binary LR was used in the analysis. Interobserver agreement for radiomic feature extraction was evaluated using the intra-class correlation coefficient (ICC).

## 3 Results

### Clinical characteristics

The study included 92 GC patients (29 HER2-positive, 63 HER2-negative) with comparable demographic and clinicopathological profiles (**Table 1**). Notably, elevated CEA levels were significantly more prevalent in HER2-positive patients (51.7% vs. 20.6%, P = 0.03). Though HER2 positivity was numerically higher in males (37.7% vs. 19.4% in females, P = 0.07), no statistically significant differences were observed in gender, age, tumor location, tumor size, CA199, or AFP levels (all P > 0.05).

**Table 1:** Demographic and clinical characteristics of the patients.

Characteristics	Total	Her2 positive	Her2 negative	P value
Gender				0.07
Male	61	23	38	
Female	31	6	25	
Age(mean ± SD, years)		67.74 ± 11.44	66.95 ± 10.13	0.63
Tumor Location				0.54
Non-cardia	78	26	52	
Cardia	14	3	11	
Size (mean ± SD, mm)		4.14 ± 1.81	4.38 ± 2.84	0.79
CEA				0.03*
CEA (-)	64	14	50	
CEA (+)	28	15	13	
CA199				0.16
CA199 (-)	66	18	48	
CA199 (+)	26	11	15	
AFP				0.53
AFP (-)	90	28	62	
AFP (+)	2	1	1	

* P < 0.05, indicating that the difference was statistically significant.

### Endoscopic and radiomic feature extraction and selection

**Figure 2:**
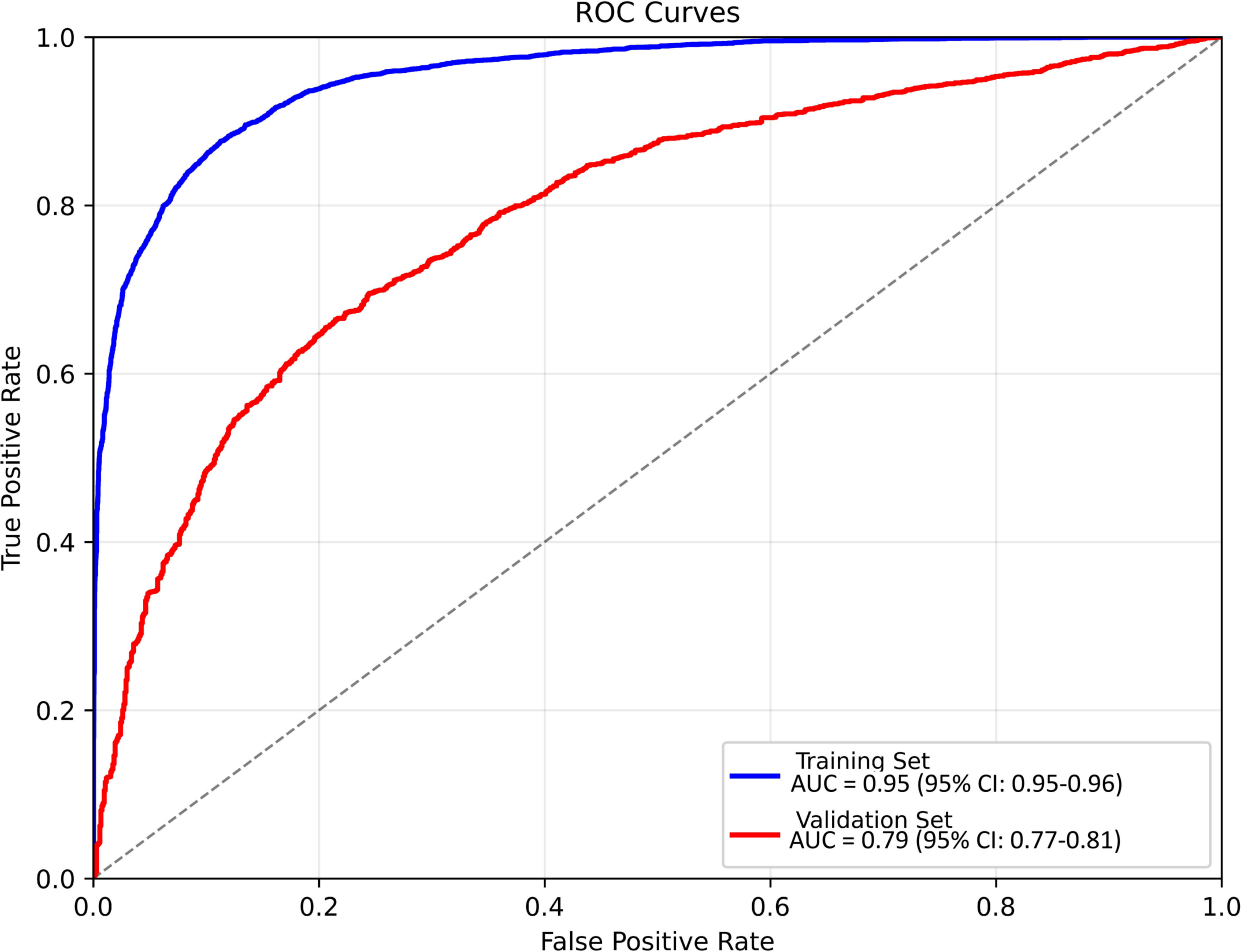
Receiver operating characteristic (ROC) curves and performance metrics of the endoscopic model for predicting gastric cancer (GC) invasion depth. In the training dataset, the model achieved an area under the ROC curve (AUC) of 0.95. In the validation dataset, the AUC was 0.78.

The DL features extracted from ResNet50’s avgpool layer across 402 GC cases were refined via LASSO regression, yielding three optimal predictors (DLfeature_1, DLfeature_2, and DLfeature_8) with non-zero coefficients. Simultaneously, the invasion depth prediction model, trained on endoscopic images, demonstrated high discriminative performance with an AUC of 0.95 (95% CI: 0.95- 0.95) in the training set and 0.79 (95% CI: 0.77-0.81) in the validation set (**Figure 2**). The additional evaluation metrics are listed in **Table 2**. Then the predicted probability of deeper invasion from this model was incorporated as a variable feature alongside the selected DL features.

**Table 2:** Performance metrics of the tumor invasion depth prediction model.

Dataset	AUC (95% CI)	Accuracy	Precision	Recall	F1 Score	Specificity
Training Set	0.95 [0.95, 0.96]	0.86	0.94	0.77	0.85	0.95
Validation Set	0.79 [0.77, 0.81]	0.68	0.89	0.60	0.72	0.83

A total of 2, 153 radiomic features were initially extracted across wavelet, logarithm, and morphological transforms. Through sequential feature selection: (1) variance thresholding removed 225 features; (2) correlation-based redundancy elimination (threshold >0.9) excluded 1, 587 features; (3) inter-observer consistency filtering (ICC >0.75) discarded 264 features; and (4) importance ranking retained the top 7 discriminative features. The final retained features were: square_glszm_SmallAreaLowGrayLevelEmphasis (Square_GLSZM_SALGE), wavelet-LHH_glcm_Imc1 (Wavelet_LHH_GLCM_Imc1), wavelet-HHL_glszm_SmallAreaHighGrayLevelEmphasis (Wavelet_HHL_GLSZM_SAHGE), logarithm_glcm_MCC (Log_GLCM_MCC), log-sigma-2-0-mm-3D_glszm_SizeZoneNonUniformityNormalized (Log_sigma-2-0_GLSZM_SZNU), wavelet-HHH_glcm_MaximumProbability (Wavelet_HHH_GLCM_MaxProb), wavelet-HHL_glszm_ZoneVariance (Wavelet_HHL_GLSZM_ZV)—were retained.

### Construction and evaluation of models

**Figure 3:**
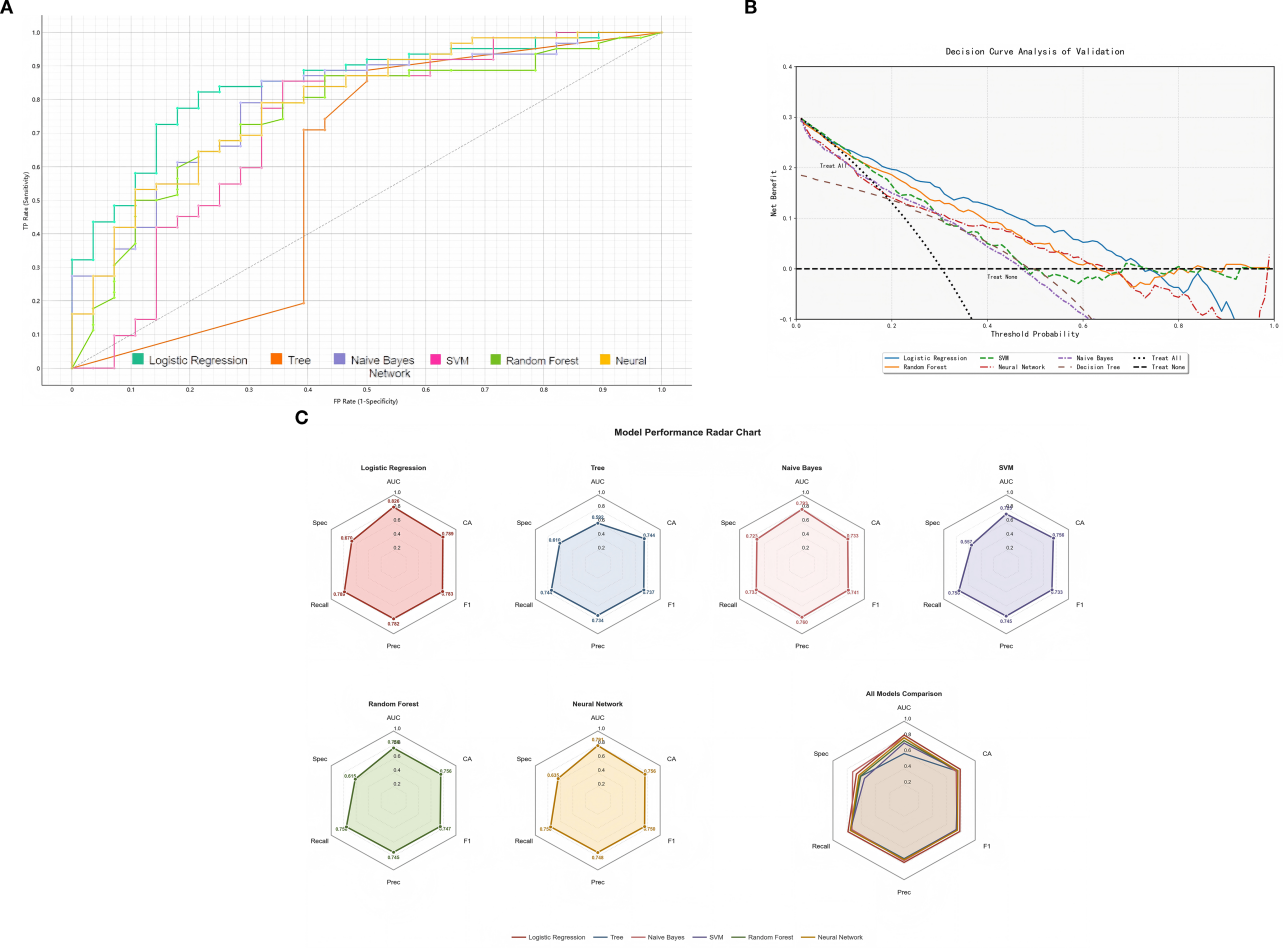
Evaluation of multiple machine learning models: **(A)** Receiver operating characteristic (ROC) curves for the six machine learning models (Decision Tree, Logistic Regression, Naive Bayes, Support Vector Machine, Random Forest, and Neural Network) based on leave-one-out cross-validation; **(B)**Decision curve analysis (DCA) of multiple machine learning models; **(C)** Radar chart for multiple machine learning models.

**Figure 4:**
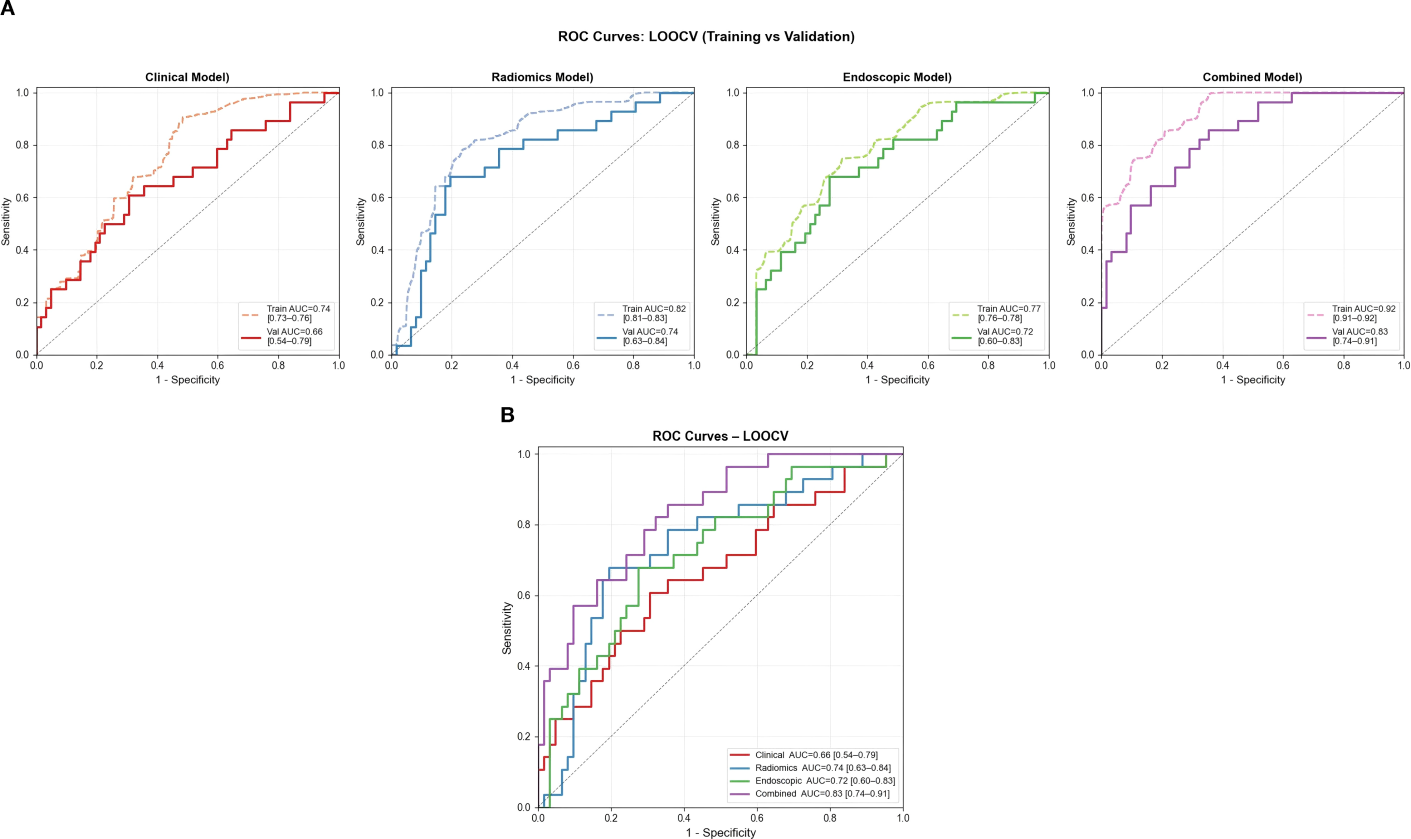
The ROC curves of the four logistic regression (LR) models (Endoscopic, Clinical, Radiomic, and Combined) based on leave-one-out cross-validation **(A, B)**.

The final HER2 status prediction model integrated multimodal features: (1) invasion depth probability, (2) three DL features (3) seven radiomic features, and (4) clinical biomarker CEA. Six machine learning algorithms were evaluated (**Figures 3A, B**). The AUC, accuracy, sensitivity, specificity, negative predictive value, and positive predictive value are listed in **Table 3** and visualized through a radar chart (**Figure 3C**). Using LR as the optimal algorithm, the results from the ROC curves in both the training and validation sets demonstrate the varying performance of the four models (Endoscopic, Clinical, Radiomic, and Combined). In the training set, the Combined model achieved the highest AUC of 0.92 (95% CI: 0.91-0.92). In the validation set, the Combined model again outperformed the others with an AUC of 0.83 (95% CI: 0.73-0.90). The AUC (95% CI) of the four models are shown in **Figures 4A, B**.

**Table 3:** Comparative performance of six machine learning models.

Model	AUC	Accuracy	Precision	Recall	F1 Score	Specificity
Tree	0.59	0.74	0.73	0.74	0.74	0.61
Logistic Regression	0.83	0.79	0.78	0.79	0.78	0.67
Naive Bayes	0.79	0.73	0.76	0.73	0.74	0.72
SVM	0.73	0.76	0.75	0.76	0.73	0.56
Random Forest	0.76	0.76	0.75	0.76	0.75	0.62
Neural Network	0.79	0.76	0.75	0.76	0.75	0.64

### The best model interpretation

**Figure 5:**
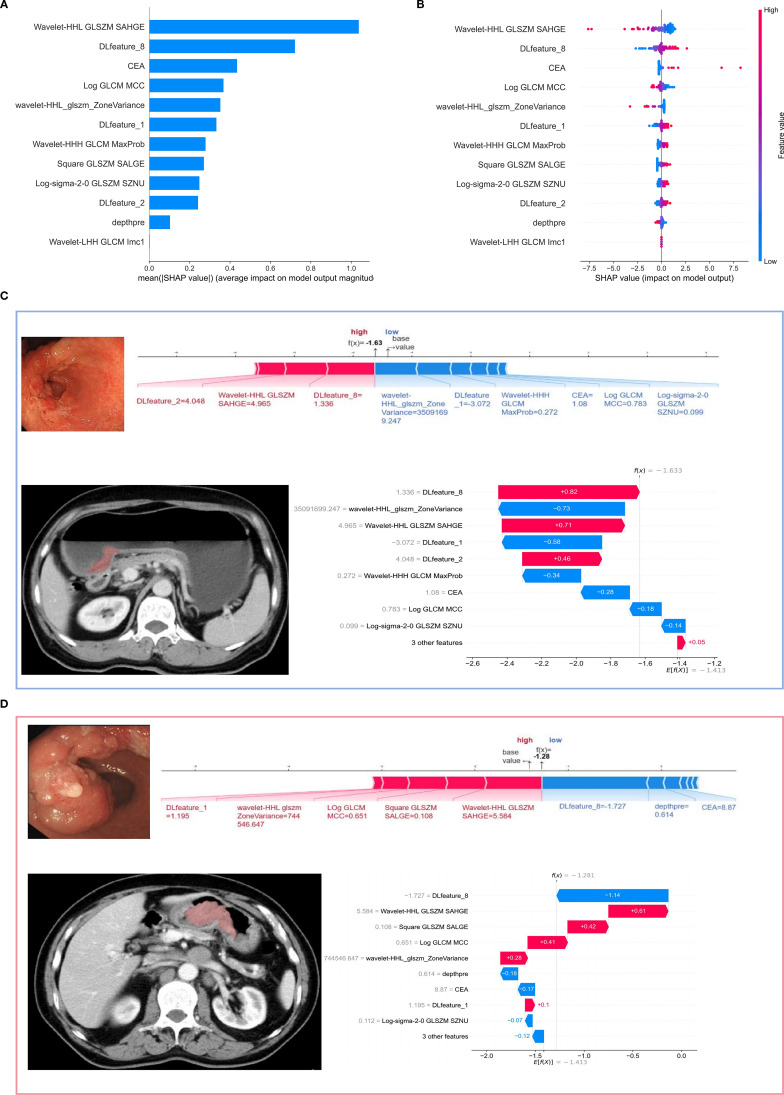
**(A)** The SHAP bar plot quantifies the global feature importance within the multimodal integration framework, ranking features by their mean absolute impact on model predictions. **(B)** The SHAP beeswarm plot illustrates the positive or negative influence of each feature on predictions, with colors representing feature values (red for high, blue for low). **(C, D)** SHAP waterfall plot and force plot. **(C)** A 54-year-old female with HER2-negative gene expression has a predicted HER2 status of -1.63, reflecting her negative expression. **(D)** A 65-year-old male with HER2-positive gene expression shows a predicted HER2 status of -1.28, consistent with his positive expression. The SHAP values are shown on the horizontal axis, and the vertical axis represents the feature values for the sample. A blue color indicates a negative influence of the feature on the prediction (arrow pointing left, SHAP value decreases), while red signifies a positive influence (arrow pointing right, SHAP value increases). At the bottom, E[f(x)] refers to the SHAP base value, representing the model’s mean prediction, with E denoting expectation. In this multimodal integration framework, E = -1.41.

The overall SHAP analysis was performed to interpret the combined model and aid in its clinical use. In the SHAP bar chart (**Figure 5A**), the relative importance of each feature is shown, with Wavelength-HHL_GLSZM_SAHGE emerging as the most influential, followed by DLfeature_8 and CEA. The SHAP beeswarm plot (**Figure 5B**) demonstrates how each feature’s value affects the prediction, with higher values (in red) generally leading to higher prediction probabilities. The SHAP force plots (**Figures 5C, D**) for two cases represent HER2 gene expression status. The first case, for HER2-positive, shows DLfeature_8 negatively impacting the prediction, while Wavelength-HHL_GLSZM_SAHGE and other features positively contribute, with a final prediction of -1.28. In the second case, for HER2-negative, DLfeature_8 increases the prediction, along with Wavelength-HHL_GLSZM_SAHGE and DLfeature_2, leading to a final prediction of -1.63.

## 4 Discussion

This study innovatively developed an interpretable hierarchical multimodal framework for HER2 status prediction in GC patients, systematically evaluating various machine learning algorithms. LR emerged as the optimal predictor, demonstrating superior performance in classifying HER2 status. Our findings emphasize the critical value of synthesizing diverse data modalities to refine preoperative assessment of HER2 expression and enhance personalized treatment strategies for GC patients.

In recent years, radiomics has shown great promise in GC research by quantitatively characterizing tumor heterogeneity from CT images and linking imaging features with molecular and clinicopathologic profiles (25–30). Building on this foundation, several studies have specifically explored CT-based radiomics for HER2 status prediction. Wang et al. established a contrast-enhanced CT radiomics model that achieved AUCs of 0.83 and 0.72 in arterial and portal phases, respectively (18). Li et al. proposed a CT-based radiomics nomogram integrating clinical variables (AUC = 0.78) (17). More recently, Zhao et al. developed and externally validated a radiomics model, reaching AUCs of 0.73 to 0.71 across cohorts. In this study, the final radiomics signature was dominated by GLSZM and GLCM features extracted from wavelet- and logarithm-filtered images. These higher-order features are robust mathematical descriptors of macroscopic tumor heterogeneity and texture roughness (31). Notably, the feature with the highest predictive contribution was Wavelet_HHL_GLSZM_SAHGE. Radiologically, SAHGE measures the concentration of smaller size zones with high gray-level values, reflecting fine, high-density focal patches within the tumor (31). In the context of contrast-enhanced CT, these high-attenuation sub-regions may correspond to areas of focal hypervascularity or dense cellular clusters. Biologically, this finding aligns with the known aggressive behavior of HER2-positive gastric cancer. Previous studies have demonstrated that HER2 overexpression is strongly correlated with tumor angiogenesis, often driven by the upregulation of vascular endothelial growth factor (32). The resulting chaotic micro-vascular networks and rapid cellular proliferation manifest as heightened intra-tumoral heterogeneity on CT images. Furthermore, other retained features, such as Log_sigma_GLSZM_SZNU and Wavelet_HHL_GLSZM_ZV, directly quantify spatial non -uniformity. High values in these features suggest a highly disorganized tumor microenvironment, likely reflecting the presence of micro-necrosis, varied stromal responses, and mixed cellular clones that are frequently observed in aggressive HER2-positive phenotypes.

Studies have also explored the relationship between GC invasion depth and HER2 expression, with some indicating that HER2 overexpression is associated with deeper tumor invasion and more aggressive cancer features (33, 34). However, other research has shown inconsistent results (35). Our findings demonstrate that CNNs can effectively predict invasion depth from endoscopic images. However, when applied to predict HER2 gene expression, the invasion depth exhibited only a minimal contribution to the model’s performance. Conversely, when CNN-derived features were extracted and integrated into the predictive model, they significantly enhanced the model’s overall prediction accuracy. These results indicate that feature-level fusion may offer superior performance compared to decision-level fusion in improving predictive outcomes.

The strength of this study lies in the integration of a hierarchical and multi-modal predictive framework to provide a more comprehensive view of the tumor’s characteristics. To the best of our knowledge, no prior studies have incorporated endoscopic images alongside radiomic and clinical variables for the prediction of HER2 expression in GC. Using ResNet50 to extract features from endoscopic images, combined with a prediction model for invasion depth, enables the identification of complex relationships often overlooked by traditional methods. CT imaging provides crucial anatomical details, and radiomics extracts high-throughput quantitative features, offering insights into tumor heterogeneity. DL further improves model performance through data augmentation and transfer learning, while facilitating cross-disciplinary collaboration, allowing both radiologists and gastroenterologists to interpret complex imaging data. This integrated approach promotes more informed decision-making and offers a holistic method for predicting HER2 expression in GC.

Previous studies have typically employed single machine learning models (18, 19, 36) whereas this study integrates multimodal features to construct models using six different machine learning algorithms. The results indicate that the LR model demonstrates superior performance in predicting HER2 gene expression in GC patients, with AUC values of 0.93 and 0.85 in the training and validation cohorts, respectively. These values are notably higher than those of other models in the validation set, and the LR model also exhibits superior robustness. The LR model is simple, interpretable, and particularly suitable for clinical scenarios requiring clear causal relationships (37, 38). It demonstrates stability and strong generalization ability (38), particularly excelling when there is a linear relationship between features and outcomes (34). Our multi-algorithm approach proved advantageous over single-algorithm methods for identifying the optimal model (LR). Within this LR framework, the combined model integrating all modalities significantly outperformed models based on individual clinical, radiomic, or endoscopic features.

Based on the SHAP analysis results from the multimodal model, the top three features contributing to the prediction of HER2 gene expression in GC are derived from three different modalities: endoscopic imaging, radiomics, and clinical data. Endoscopic features provide detailed visual insights into the tumor’s surface morphology, radiomic features capture in-depth patterns from medical images, and clinical data such as CEA levels offer physiological insights. By integrating these distinct data sources, the model can leverage both the spatial and non-spatial information, increasing its ability to predict HER2 gene expression accurately. Two representative cases (**Figures 5C, D**) further validate the model’s clinical applicability. Both the tumor heterogeneity-associated Wavelength-HHL_GLSZM_SAHGE feature and DLfeature_8 ranked among the top three most important predictors, with final predictions concordant with definitive pathological diagnoses. These clinically aligned results underscore the model’s quantitative predictive capability.

Our study had some limitations. First, Selection bias could have been introduced due to the retrospective design. Second, the limited sample size in our dataset may have restricted the model’s ability to generalize, particularly given the relatively low prevalence of HER2-positive cases. Third, the quality of the endoscopic images—subject to variations in equipment, operator technique, and imaging conditions—may have introduced noise and reduced the overall informativeness of this modality. Additionally, the data was derived from a single-center dataset, which may limit applicability across different scanners and imaging protocols. Finally, hyperparameters were not extensively tuned. Future work should therefore emphasize the inclusion of larger, more diverse datasets, external validation cohorts, and potentially multimodal data integration to improve the robustness and translational value of predictive models.

In conclusion, we established a artificial intelligence framework integrating multi-source data for HER2 assessment in GC. This integrated framework highlights the clinical value of synthesizing endoscopic, radiomics, and clinical data for personalized HER2 status assessment, providing a powerful tool to guide individualized targeted therapies.

## Data Availability

The raw data supporting the conclusions of this article will be made available by the authors, without undue reservation.
