# Supplementary material for: Interpretable deep learning-based hierarchical multi-modal fusion model for predicting HER2 expression in gastric cancer
Source: Front Oncol. 2026 May 18;16:1745228. doi: 10.3389/fonc.2026.1745228 (PMC13223084; doi:10.3389/fonc.2026.1745228)
Supplement: Supplementary file 1 [file Table1.docx]

**Table S1. Key hyperparameters of six machine-learning classifiers implemented in Orange**

| Model | Key hyperparameters (Orange widget settings) |
| --- | --- |
| Decision Tree | Induce binary tree = **True**; Min. number of instances in leaves = **2**; Do not split subsets smaller than = **5**; Limit maximal tree depth = **100**; Stop when majority reaches = **95%** |
| Logistic Regression | Regularization type = **Ridge (L2)**; **C = 1**; Balance class distribution = **False** |
| Naive Bayes | **Default settings** (no tunable parameters exposed in Orange widget) |
| SVM | Type = **C-SVM**; Kernel = **RBF**; **C = 1.0**; **gamma (g) = auto**; Numerical tolerance = **0.001**; Iteration limit = **100** |
| Random Forest | Number of trees = **10**; “Number of attributes considered at each split” = **not enabled (default)** ; Limit depth of individual trees = not enabled (default); Do not split subsets smaller than = **5**; Replicable training = **True**; Balance class distribution = **False** |
| Neural Network | Hidden layers = **(100)**; Activation = **ReLU**; Solver = **Adam**; Regularization **α = 0.0001**; Max iterations = **200**; Replicable training = **True** |
